# Pyrazine analogs are active components of wolf urine that induce avoidance and fear-related behaviors in deer

**DOI:** 10.3389/fnbeh.2014.00276

**Published:** 2014-08-14

**Authors:** Kazumi Osada, Sadaharu Miyazono, Makoto Kashiwayanagi

**Affiliations:** ^1^Division of Physiology, Department of Oral Biology, School of Dentistry, Health Sciences University of HokkaidoIshikari-Tobetsu, Hokkaido, Japan; ^2^Department of Sensory Physiology, Asahikawa Medical UniversityAsahikawa, Hokkaido, Japan

**Keywords:** pyrazine analog, wolf, Hokkaido deer, field bioassay, avoidance, fear, repellent, kairomone

## Abstract

Our previous studies indicated that a cocktail of pyrazine analogs, identified in wolf urine, induced avoidance and fear behaviors in mice. The effects of the pyrazine cocktail on Hokkaido deer (*Cervus nippon yesoensis*) were investigated in field bioassays at a deer park in Hokkaido, Japan. A set of feeding bioassay trials tested the effects of the pyrazine cocktail odor on the behavior of the deer located around a feeding area in August and September 2013. This odor effectively suppressed the approach of the deer to the feeding area. In addition, the pyrazine cocktail odor provoked fear-related behaviors, such as “tail-flag”, “flight” and “jump” actions, of the deer around the feeding area. This study is the first experimental demonstration that the pyrazine analogs in wolf urine have robust and continual fearful aversive effects on ungulates as well as mice. The pyrazine cocktail might be suitable for a chemical repellent that could limit damage to forests and agricultural crops by wild ungulates.

## Introduction

Wild animals frequently infiltrate human habitats, where they can cause serious trouble. For example, the damage that deer cause to agricultural, horticultural, and forest resources is an economic problem not only in Hokkaido (Masuko et al., 2011) but around the world (Trdan et al., 2003; Killian et al., 2009; Kimball et al., 2009; Baasch et al., 2010; Gheysen et al., 2011). Rather than eliminating deer, it is ideal to control their behavior so that they coexist with wild animals without destroying human habitats and natural environments.

The detection of predator phenotypic traits by prey species is a vitally important function of communication among mammals. How prey discerns a predator remains to be elucidated; it most likely involves a range of sensory and behavioral signals. For animals that rely on chemical communication to regulate social and sexual interactions, there is some indication that the presence of a predator can be detected by its scent. When the recipient benefits from the signal, the molecules involved are called kairomones (Wyatt, 2003; Rodriguez, 2010).

Many studies have shown that the odors of a predator induce avoidance and fear in various kinds of herbivores. For instance, black-tailed deer (*Odocoileus hemionus columbianus*) and/or white-tailed deer (*Odocoileus virginianu*s) aversively respond to the odor of the urine of several predators, including wolf (*Canis lupus*), coyote (*Canis latans*), fox (*Vulpes* *vulpes*), wolverine (*Gulo gulo*), lynx (*Lynx canadensis*), and bobcat (*Lynx rufus*), as well as to the odor of the feces of cougar (*Puma concolor*), coyote, and wolf (Sullivan et al., 1985b; Swihart et al., 1991). Similarly, odors emitted by several kinds of predators induce defensive behaviors in hare (*Lepus americanus*) (Sullivan et al., 1985a) and experimental rats (*Rattus norvegicus*) (Fendt, 2006). Moreover, American beaver (*Castor canadensis*), cattle (*Bos taurus*), and marsupials that are exposed to the odor of wolf or dingo (*Canis lupus dingo*) showed defensive or avoidance responses (Lindgren et al., 1995; Kluever et al., 2009; Parsons and Blumstein, 2010). Those studies clearly indicate that many carnivores’ urine and feces including wolf contain kairomones, which repel their prey animals. As a practical matter, predator wolf urine is used to drive away these animals without killing them (Sullivan et al., 1985a,b; Lindgren et al., 1995; Severud et al., 2011).

According to our recent study (Osada et al., 2013), urine odors of the common gray wolf induce aversive and fear-related responses in mice in an experimental setting. In addition, these responses are caused mainly by the presence of certain volatile pyrazine compounds, namely 2,6-dimethyl pyrazine (DMP), trimethyl pyrazine (TMP), and 3-ethyl-2,5-dimethyl pyrazine (EDMP), in wolf urine. The cocktail of DMP, TMP, and EDMP (pyrazine cocktail) is more potent than any one component alone. These pyrazine analogs, which retain characteristic roasted aromas in various foods, are known as safe compounds with no carcinogenicity and with low acute toxicity (EFSA Panel on Food Contact Materials, Enzymes, Flavourings and Processing Aids (CEF), 2011). Actually, some of alkylpyrazines are widely used in the food industry as a flavor ingredient (Burdock and Carabin, 2008). Therefore, the pyrazine analogs are expected to be favorable herbivore repellents without destroying the natural habitat and agriculture.

We hypothesized that the pyrazine analogs, odors of predator wolf, are at least a portion of the putative kairomones that induce avoidance and fear in various prey species. In this study, we explored the effects of pyrazine analogs to Hokkaido deer (*Cervus nippon yesoensis*), a kind of large herbivores. The analogs were found to act as repellents for deer and also to directly elicit fear-related reactions in deer, such as “tail-flag”, “flight” and “jump” (Caro, 2005; Stankowich and Coss, 2006). The present results suggested that the pyrazine analogs provoke aversion and fear not only in mice but also in large herbivores.

## Materials and methods

### Study area

The field work was conducted in a deer park (44°12’ N and 142°48’ E, Nishiokoppe, Hokkaido, Japan), a commercial wildlife park located within a reservation and conservation area. Over 30 Hokkaido deer inhabited an enclosed area of more than 9 ha. They had free access to herbage, bamboo grass, tree leaves and bark, and water located in the park. All of them were considered healthy. They were sometimes fed with steam-flaked corn, whose chemical composition was crude protein 7.6%, ether extract 3.8%, crude fiber 1.7%, crude ash 1.2%, nitrogen-free extract 71.3%, and moisture 14.5% (Hokuren Federation of Agricultural Cooperatives, Hokkaido, Japan).

### Experimental design

**Figure 1:**
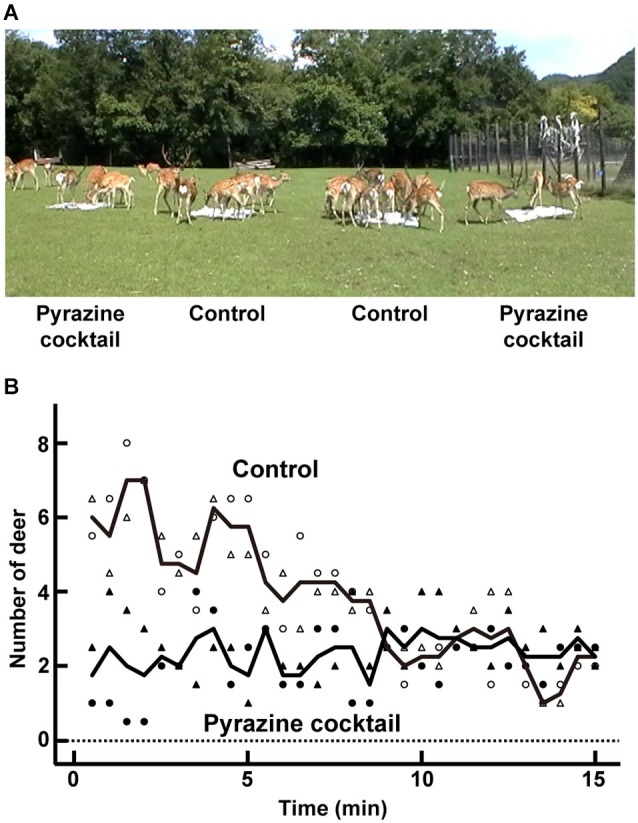
**Feeding trial. (A)** An average of 24 deer participated in the feeding trial at the deer park in Nishiokoppe, Hokkaido. The pyrazine cocktails were placed in two of the four feeding areas. **(B)** Average changes in the number of deer surrounding the pyrazine cocktail (closed symbols) or control feeding area (open symbols). The numbers were plotted by counting the deer near in each feeding area every 30 s. Symbols of circle and triangle indicate the average number from two feeding areas per odor condition in the trials in August and September, respectively. Lines show the average values from the both trials.

The study was carried out in accordance with the Guidelines for the Use of Laboratory Animals of the Asahikawa Medical University and approved by the Nishiokoppe collegium of deer nurturing (NOP-130708). 2,6-Dimethyl pyrazine (DMP) and TMP were purchased from Tokyo Chemical Industry (Tokyo, Japan), and 3-ethyl-2,5-dimethyl pyrazine (EDMP) was purchased from Alfa Aesar (Ward Hill, MA, USA). Feeding bioassay trials (Figure 1A) were carried out twice, on 27 August and 19 September, 2013. The deer in the trial included 12 males and 10 females in August, and 16 males and 9 females in September. The basic design of the bioassay trial in this study utilized square translucent sheets (1.8 m × 1.8 m) with food and odor sources. The four sheets with 5 kg of steam-flaked corn put on each of the center (feeding area) were placed at approximately 3 m intervals on a line. In order to prevent the animals from accidentally destroying the odor sources, self-made odor generators were constructed from iron tubes (2.5 cm i.d. × 25 cm length equipped with 40 odor holes, each having a diameter of 5 mm), into which were inserted 2 ml pyrazine cocktail (DMP, TMP, and EDMP, 33% v/v of each) or no odorant (control) mixed with cotton. At two of the four feeding areas, the odor generators containing the pyrazine cocktail were put on each of four corners (that is, 8 ml pyrazine cocktail per feeding area), and the others were left with the control odor generators. An animal’s movements and behaviors were recorded by two observers, each with a video camera, positioned 10 m away (a distance that did not interfere with the animal’s behavior). The trials were terminated after 15 min.

### Data collection and processing

We collected and analyzed data from adults and juveniles (>1 year old) by distinguishing the sex and age of the deer according to antler and body size. Because of their inconsistent participation (just a few seconds) in the trials, we ignored three of the fawns (all three were <1 year old). We conducted the several behavioral observations for total number of 47 deer (28 males and 19 females). Movements of individual deer were evaluated by identifying its position every 2.5 s as recorded by the video camera. The positions were defined according to five position indexes: the animal pressed its head into the sheet of the control (+2) or pyrazine cocktail (−2) area; the animal was within 1 m of the feeding area but did not press its head to the sheet (control, +1; pyrazine cocktail, −1); the animal was far from the feeding area (0). We defined ±2 and ±1 of the position index as “access” and “approach”, respectively, and then quantified the avoidance behaviors from the position index traces. In addition, we noticed that some deer lifted up their tail upon accessing the feeding sheet (tail-flag), rapidly escaped with their neck retracted (flight), and sprang back (jump) from the feeding sheet associated with the pyrazine cocktail odor generators. Therefore, we recorded these reactions as behavioral measures that might indicate fear (Caro, 2005; Stankowich and Coss, 2006). An observer who was not aware of each animal’s test condition later analyzed each deer’s movements and behaviors as recorded on video.

### Statistical analysis

Data are given as means ± SEM. Overall statistical differences were determined using Friedman tests for changes in the duration and frequency of access. Differences between the pyrazine cocktail and control areas were detected using Wilcoxon signed-rank tests for paired time periods. Differences between males and females were tested by Mann-Whitney *U*-tests. The criterion for statistical significance was *p* < 0.05 in all cases.

## Results

### Pyrazine analog-induced suppression of deer approach feeding area

To explore the avoidance effect of pyrazine analogs on deer, we conducted feeding trials in August and September (Figure 1A). During the first 5 min, the average numbers of deer attracted to the feeding areas were approximately two and six by the presence and absence, respectively, of a pyrazine cocktail (Figure 1B). The poor attraction of the feeding area pervaded by the pyrazine cocktail odor remained until the end of the 15-min trial (Figure 1B). This result indicates that the odor of pyrazine analogs may inhibit deer from approaching despite the presence of maize.

### Avoidance behaviors elicited by odor of pyrazine analogs

**Figure 2:**
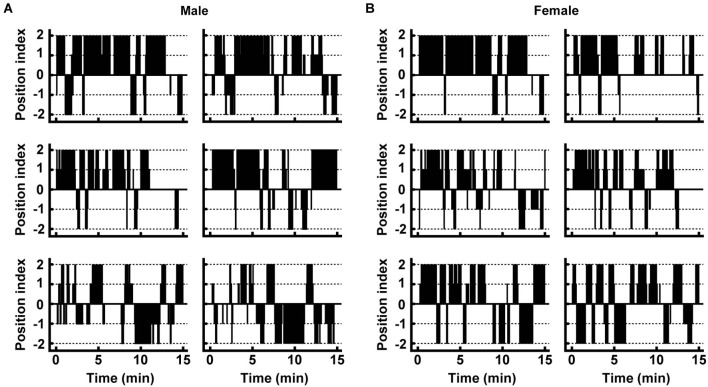
**Movement of individual male and female deer in the trial. (A,B)** Typical movements are shown for males and females. Plots of the position index were made at every 2.5-s time point. Positive and negative numbers of the position index indicate the presence of the individual near the control and pyrazine cocktail feeding areas, respectively (see Section Materials and Methods for details).

In order to examine the effect of the pyrazine cocktail on individual deer, we first evaluated the movements of the individuals (see details in Section Materials and Methods). Among the 28 males and 19 females participating in the two trials, most of them spent more time eating maize grain in the control feeding area than in the pyrazine cocktail area (Figures 2A,B).

**Figure 3:**
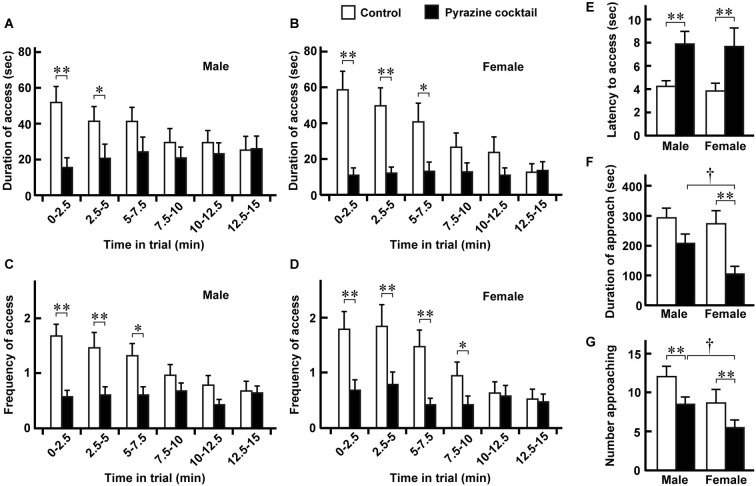
**Avoidance behaviors of male and female deer. (A,B)** Duration of access was defined as the length of time that males (*n* = 28) and females (*n* = 19) spent putting their heads to the sheet in either feeding area. Means were obtained from the individual animals during every 2.5-min period of the 15-min trial. **(C,D)** Frequency of access was defined as the number of times that males and females craned their necks to the sheet in either area. Data were obtained from the same deer pack as in **(A)** and **(B)**. **(E)** Latency to the access from the approach. Data were obtained from the same deer pack except for one male that had no access. **(F,G)** Duration **(F)** and number **(G)** of approaches to the feeding area. The means were estimated from the same pack as in **(A)** and **(B)**. “Access” and “approach” were defined as ±2 and ±1 of the position indexes shown in Figure 2. Open and closed bars indicate the control and pyrazine cocktail areas, respectively, in all panels. The time-dependent differences in the values for the pyrazine cocktail in **(A–D)** are not significant (*p* > 0.05, Friedman test). * *p* < 0.05, ** *p* < 0.01, Wilcoxon signed-rank test. ^†^ *p* < 0.05, Mann-Whitney *U*-test.

From the movement traces, we quantified avoidance behaviors at the pyrazine cocktail and control odor feeding areas. Between the trials in August and September, there were no dramatic differences in any of the avoidance or fear-related behaviors (described below) of deer at the feeding areas (*p* > 0.05, Mann-Whitney *U*-test; Supplementary Figure S1). We then compared avoidance behaviors between males and females (Figure 3). Both males and females spent less time in the pyrazine cocktail area than in the control area during the first 5 min of the trial, and this was also the case throughout the 15-min trial (Figures 3A,B). The changes in the frequency of access were also similar to those in the duration (Figures 3C,D). Moreover, for both sexes, the odor of the pyrazine cocktail increased the latencies to reach the feeding area from their approach within 1 m (Figure 3E). Interestingly, females showed poorer approaches to the feeding area than males in the presence of the pyrazine cocktail (Figures 3F,G). These results indicate that both males and females avoid pyrazine cocktail odor and do not become easily habituated to the odor for tens of minutes.

### Fear-related behaviors provoked by pyrazine analogs

**Figure 4:**
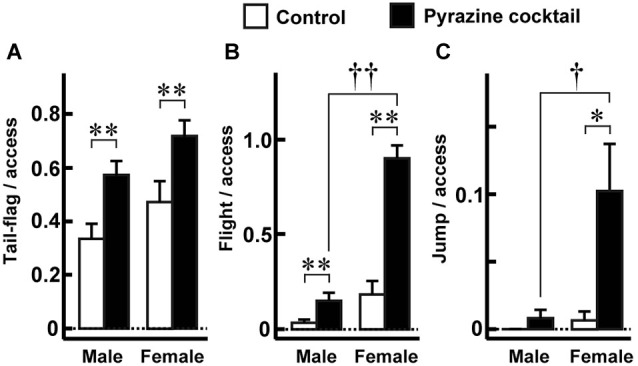
**Fear-related behaviors during access to the feeding area**. **(A–C)** The proportions of tail-flag **(A)**, flight **(B)**, and jump **(C)** actions were made by measuring the time of each behavior by males (*n* = 27) and females (*n* = 19) during the 15-min trial. The values were normalized to the numbers accessing the areas throughout the trial. Open and closed bars indicate control and pyrazine cocktail areas, respectively, in all panels. * *p* < 0.05, ** *p* < 0.01, Wilcoxon signed-rank test. ^†^ *p* < 0.05, ^††^ *p* < 0.01, Mann-Whitney *U*-test.

Since the pyrazine cocktail provokes fear-related behaviors in mice (Osada et al., 2013), we examined whether the pyrazine cocktail could induce these behaviors in deer. We quantified the tail-flag, flight, and jump actions, which are known to be fear responses of deer to predators (Caro, 2005; Stankowich and Coss, 2006). In both sexes, tail-flag was observed more frequently during access to the pyrazine cocktail feeding than during access to the control area feeding (Figure 4A). The other fear responses, flight and jump, were observed mainly in females (Figures 4B,C). These results indicate that the odor of the pyrazine cocktail could provoke fear in deer.

## Discussion

The present study shows that Hokkaido deer are repelled by the odor of a cocktail of pyrazines identified in wolf urine, and that this odor in a feeding area significantly inhibits their approach to the area (Figure 1). In addition, in order to explore the individual deer behaviors, the pyrazine cocktail odor’s ability to keep deer from entering foraging areas were clarified (Figures 2–4). Moreover, these effects were observed similarly at least 1 month after the first experiment day (Supplementary Figure S1). As mentioned in the Introduction section, we recently clarified that wolf urine odors induce aversive and fear-related responses in mice in an experimental setting (Osada et al., 2013). In this paper, we clarified that these activities were mainly due to the presence of certain volatile pyrazine compounds. Previous studies identified novel kairomones from odor sources of predators of rodents (Vernet-Maury et al., 1984; Wallace and Rosen, 2000; Papes et al., 2010; Ferrero et al., 2011). However, we did not find any reports that confirmed the effects of these kairomones on other kinds of mammals, including ungulates.

Previous studies clearly indicated that wolf urine contains semiochemicals that repel their prey species (Jorgenson et al., 1978; Raymer et al., 1984; Sullivan et al., 1985a,b; Nolte et al., 1994). Although there are numerous studies about repellents to ungulates, we are not aware of any that identified effective kairomone(s) to ungulates. Of these, Δ^3^-isopentenylmethyl sulphide and its derivatives are candidate predator kairomones (Wilson et al., 1978). However, their capacity to provoke avoidance behaviors in ungulates is limited (Wilson et al., 1978; Hani and Conover, 1995; Lindgren et al., 1995). Therefore, to the best of our knowledge, a mixture of pyrazine analogs is the first example of kairomones that provoke an aversive effect in both rodents and ungulates. However, we do not preclude the significance of the above-mentioned putative kairomones previously identified. In addition, a synergistic effect might exist between pyrazine analogs and these alkyl sulfides.

In the present study, we observed that the proportions of fear-related behaviors, such as tail-flag, flight, and jump were significantly higher in the presence of the pyrazine cocktail than of the control. Interestingly, some of these fearful behaviors depended on sex; remarkably, female but not male deer exhibited fearful reactions (Figure 4). According to a previous study on experimental mice, the magnitude of avoidance of trimethylthiazoline (TMT) as well as of natural fox feces was significantly higher in female than in male mice (Buron et al., 2007). Moreover, Perrot-Sinal et al. (1996) provided evidence for sex differences of meadow voles in both basal activity level and activity following exposure to the odor of a predator red fox. Therefore, this present result shows that ungulates also exhibit sex-differences in avoidance behavior.

The extent to which Hokkaido deer remain averse to pyrazine analogs over time remains to be seen. In this study, we clarified that the avoidance effects provoked by pyrazine analogs were observed even 1 month after the first application of the pyrazine cocktail to deer belonging to the same pack (Supplementary Figure S1). This implies that pyrazine analogs maintain their effect on deer over time. A few previous studies demonstrated the continual effectiveness of wolf urine as a repellent. Parsons and Blumstein (2010) demonstrated that, despite repeated exposure to the scent of dingo, macropodids persistently avoided an area of highly palatable food. In addition, Sullivan et al. (1985b) demonstrated that the effectiveness of wolf urine odor in suppressing the feeding of black-tailed deer on salal was significantly more effective than a control for at least 6 days. Therefore, it is conceivable that the pyrazine analogs are at least a portion of the components that evoke the significant and prolonged aversive effect of wolf urine on prey animals. Actually, in our preliminary experiment on mice, the analogs showed a powerful effect by repeated exposures (data not shown). In the present study, we have conducted the trials on two occasions. Obviously, further experimental study is needed to determine whether the odor of pyrazine analogs has continual aversive effects for extended periods to deer.

Our observations also raise the question of why Hokkaido deer avoid pyrazine analogs even though Japanese wolf (*Canis lupus hodophilax*), a potential predator, has been extinct for about 100 years (Ministry of the Environment, 2014). The extinction of a large carnivore as a consequence of anthropogenic disturbance induces important changes in ecological patterns involving behavior and interspecific ecological interactions (Berger, 1999). Actually, Pyare and Berger (2003) demonstrated that female moose (*Alces alces*) from a region (Mainland Alaska) with wolves and grizzly bear (*Ursus arctos*) assemblage responded significantly more strongly to odors of both carnivores more than did female moose from Grand Teton National Park (Wyoming), where these predators had been absent for 60–75 years until the 1990s. Therefore, it is conceivable that our present results are at odds with the previous results. However, they also found that the vigilance behavior of Mainland Alaska moose to wolf odor was significantly higher than that of Wyoming moose, but surprisingly was not higher than that of moose in a predator-free region (Kenai Peninsula) population, suggesting that learning was not a necessary component of wolf urine avoidance (Pyare and Berger, 2003). Moreover, a recent study demonstrated that black tail deer react more strongly to wolf cues than to cues associated with the less dangerous black bear (*Ursus americanus*), despite having had no contact with wolves for more than 100 years (Chamaillé-Jammes et al., 2014). Therefore, the present results suggested that pyrazine analogs are at least one of the components that provoke prey on an instinctive level. Kimball et al. (2009) indicated that avoidance of blood and other animal-derived substances may be the result of an “evolutionary memory” (Provenza, 1995) that conveys information about potential sources of pathogens. Similarly, pyrazine analogs might have conveyed information about predator odor to the prey even if the prey had never encountered that species of predator.

The prey animals detect predator odors via the main olfactory and/or the vomeronasal systems (reviewed in Takahashi, 2014). Naïve rats and mice exposed to the odor of foxes or TMT, the most effective fear-inducing component in fox feces, showed species-specific defensive responses, such as freezing in place (Vernet-Maury et al., 1984; Wallace and Rosen, 2000; Fendt et al., 2005; Buron et al., 2007; Fendt and Endres, 2008; Janitzky et al., 2009). TMT is mainly detected by the main olfactory system (Kobayakawa et al., 2007). Ferrero et al. (2011) reported that 2-phenylethylamine (2-PEA), a common constituent of carnivore urine, triggers hard-wired aversion via the olfactory sensory neurons. On the other hand, rodents exposed to cat-derived odors demonstrated fear-related responses and the elevation of stress hormones (Takahashi et al., 2005, 2007, 2008) via the accessory olfactory bulb (AOB) in the vomeronasal system (Staples et al., 2008). Papes et al. (2010) demonstrated that derivatives of major urinary proteins of rat and cat activate the vomeronasal organ and AOB neurons, and initiate defensive behaviors in mice. In a previous study, we showed that wolf urine and the volatile pyrazine cocktail also stimulate the murine vomeronasal system (Osada et al., 2013). Therefore, pyrazine analogs induce avoidance and freezing behaviors via stimulation of the murine vomeronasal system and perhaps of the main olfactory system as well. Artiodactyla, including deer (Park et al., 2014), have both olfactory systems, as do mice, suggesting that deer also detect pyrazine analogs via olfactory systems similar to those of mice. Previous reports found that TMT and 2-PEA, which induce avoidance and freezing behaviors in rodents, increase plasma corticosterone level (Kobayakawa et al., 2007; Ferrero et al., 2011). In deer, the stress level could be evaluated by measuring fecal glucocorticoid level (Millspaugh and Washburn, 2004). Further studies are required on this point.

Although the present study was conducted in a semi-natural experimental setting, we have clearly illustrated that (1) pyrazine analogs identified in wolf urine provoke an aversive effect in not only mice but also an ungulate, Hokkaido deer; (2) fear-related behaviors as well as avoidance behaviors were observed in deer; and (3) the effects of pyrazine analogs were reproduced 1 month after the first precursor experiment, suggesting the continuity of the aversive effects of the pyrazine analogs in these Hokkaido deer.

This report describes the first experimental demonstration that wolf urine kairomones, pyrazine analogs, have a robust and continual aversive effect on ungulates. However, further studies are needed in order to confirm whether pyrazine analogs provoke an aversive effect on other kinds of wild animals.

## Author contributions

Makoto Kashiwayanagi and Kazumi Osada designed the experiment. Makoto Kashiwayanagi, Kazumi Osada, and Sadaharu Miyazono performed the experiment. Sadaharu Miyazono and Makoto Kashiwayanagi analyzed the data. Kazumi Osada and Sadaharu Miyazono wrote the first draft of the manuscript. Makoto Kashiwayanagi critically revised the manuscript and all authors approved the final version.

## Conflict of interest statement

The authors declare that the research was conducted in the absence of any commercial or financial relationships that could be construed as a potential conflict of interest.
