# Supplementary material for: Pyrazine analogs are active components of wolf urine that induce avoidance and fear-related behaviors in deer
Source: Front Behav Neurosci. 2014 Aug 14;8:276. doi: 10.3389/fnbeh.2014.00276 (PMC4132518; doi:10.3389/fnbeh.2014.00276)
Supplement: Supplementary file 1 [file Data_Sheet_1.DOCX]

***Supplementary Material***

**Pyrazine analogues are active components of wolf urine that induce avoidance and fear-related behaviors in deer**

Kazumi Osada^1†^, Sadaharu Miyazono^2†^ and Makoto Kashiwayanagi^2*^

^1^Division of Physiology, Department of Oral Biology, School of Dentistry, Health Sciences University of Hokkaido, Ishikari-Tobetsu, Hokkaido, Japan

^2^Department of Sensory Physiology, Asahikawa Medical University, Asahikawa, Hokkaido, Japan

***Correspondence**:

Makoto Kashiwayanagi

Asahikawa Medical University, Department of Sensory Physiology

Midorigaoka Higashi 2-1-1-1, Asahikawa, Hokkaido 078-8510, Japan

E-mail: yanagi@asahikawa-med.ac.jp

^†^These authors have contributed equally to this work.

**Supplementary Figure**

**

**

**Supplementary Figure S1. Comparison of avoidance and fear-related behaviors between August and September.** **(A-E)** Indications of avoidance behaviors are the duration of access **(A)**, the number accessing **(B)**, the latency to access **(C)**, the duration of approach **(D)** and the number approaching **(E)**. ‘Access’ and ‘approach’ were defined as ±2 and ±1 of the position indexes shown in Figure 2, respectively. **(F-H)** Indications of fear-related behaviors are the proportions exhibiting tail-flag **(F)**, flight **(G)**, and jump **(H)** behaviors normalized to the number accessing. The data shown in this figure are made by separating the data shown in Figures 3 and 4. Open and closed bars indicate control and pyrazine cocktail areas, respectively. The number of animals is shown at each data point. The differences in the pyrazine cocktail values in all panels are not significant between August and September (*p* > 0.05, Mann-Whitney U-test).
